# Evaluating ChatGPT, Gemini and other Large Language Models (LLMs) in orthopaedic diagnostics: A prospective clinical study

**DOI:** 10.1016/j.csbj.2024.12.013

**Published:** 2024-12-26

**Authors:** Stefano Pagano, Luigi Strumolo, Katrin Michalk, Julia Schiegl, Loreto C. Pulido, Jan Reinhard, Guenther Maderbacher, Tobias Renkawitz, Marie Schuster

**Affiliations:** a Department of Orthopaedic Surgery, University of Regensburg, Asklepios Klinikum, Bad Abbach, Germany; b Freelance health consultant & senior data analyst, Avellino, Italy; c Department of Orthopaedics Hospital of Trauma Surgery, Marktredwitz Hospital, Marktredwitz, Germany

**Keywords:** Large Language Models (LLMs), GPT-4o, ChatGPT, Gemini, Llama, Gemma 2, Mistral-Nemo, Hip osteoarthritis, Knee osteoarthritis, Diagnostic sensitivity, Musculoskeletal disorders, Orthopaedic diagnostics, Patient-reported data, Artificial intelligence in healthcare

## Abstract

**Background:**

Large Language Models (LLMs) such as ChatGPT are gaining attention for their potential applications in healthcare. This study aimed to evaluate the diagnostic sensitivity of various LLMs in detecting hip or knee osteoarthritis (OA) using only patient-reported data collected via a structured questionnaire, without prior medical consultation.

**Methods:**

A prospective observational study was conducted at an orthopaedic outpatient clinic specialized in hip and knee OA treatment. A total of 115 patients completed a paper-based questionnaire covering symptoms, medical history, and demographic information. The diagnostic performance of five different LLMs—including four versions of ChatGPT, two of Gemini, Llama, Gemma 2, and Mistral-Nemo—was analysed. Model-generated diagnoses were compared against those provided by experienced orthopaedic clinicians, which served as the reference standard.

**Results:**

GPT-4o achieved the highest diagnostic sensitivity at 92.3 %, significantly outperforming other LLMs. The completeness of patient responses to symptom-related questions was the strongest predictor of accuracy for GPT-4o (p < 0.001). Inter-model agreement was moderate among GPT-4 versions, whereas models such as Llama-3.1 demonstrated notably lower accuracy and concordance.

**Conclusions:**

GPT-4o demonstrated high accuracy and consistency in diagnosing OA based solely on patient-reported questionnaires, underscoring its potential as a supplementary diagnostic tool in clinical settings. Nevertheless, the reliance on patient-reported data without direct physician involvement highlights the critical need for medical oversight to ensure diagnostic accuracy. Further research is needed to refine LLM capabilities and expand their utility in broader diagnostic applications.

## Graphical Abstract

**Figure:**
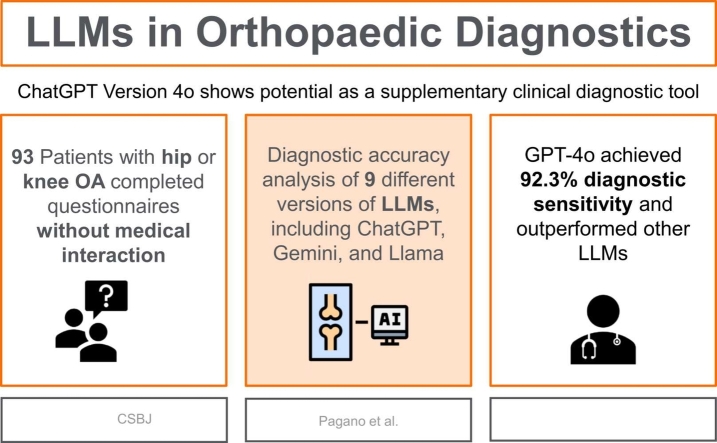

## 1 Introduction

Large Language Models (LLMs) are sophisticated algorithms designed to process and generate textual and audiovisual content. These tools have gained widespread public attention, particularly after OpenAI (San Francisco, California, US) launched ChatGPT in November 2022. Within five days, ChatGPT amassed over one million users, and by April 2023, it had achieved 1.8 billion monthly accesses [1], [2]. In May 2024, OpenAI introduced GPT-4o, a significantly enhanced version featuring improvements in processing speed, performance, and multilingual capabilities [3].

Beyond ChatGPT, other LLMs have emerged with diverse architectures and applications, including in healthcare. For instance, Gemini (Google, Mountain View, CA, USA) integrates text and multimodal processing to enhance contextual understanding [4]. Gemini has demonstrated robust capabilities in medical applications, such as multimodal reasoning, long-context comprehension, and outperforming human experts in tasks like medical text summarization [5]. Alongside cloud-based models like Gemini—which require an internet connection and pose potential regulatory challenges in data privacy, particularly in the EU [6]—other LLMs prioritize local data processing. Models such as Llama (Meta, Menlo Park, CA, USA), Gemma (Google, Mountain View, CA, USA), and Mistral-Nemo (Mistral AI, Paris, France) are designed for efficient deployment on user hardware, offering enhanced data privacy and control over computational resources [7]. Together, these models represent a spectrum of natural language processing approaches, making them invaluable for evaluating diagnostic applications.

Despite their advancements, LLMs continue to face challenges, particularly in reliably sourcing information. A notable issue is the generation of inaccurate outputs, commonly referred to as "hallucinations" [8]. This raises critical concerns regarding their trustworthiness and reliability, especially in sensitive domains like healthcare [9]. Hallucinations may present plausible but incorrect medical information, posing significant risks when patients rely on such outputs without consulting healthcare professionals [10], [11], [12].

While these models often exhibit reasoning capabilities that mimic human thought processes [13], questions persist about their dependability and safety in critical fields such as healthcare [14]. Nevertheless, LLMs hold significant potential in medical applications, including literature review, medical education, and patient data processing [15]. The integration of LLM-based tools into clinical practice has also spurred debates about their ethical implications. Key concerns include ensuring alignment with patient values, promoting fairness, mitigating biases, and adhering to robust ethical guidelines, regulatory oversight, and transparency [1], [16], [17].

In orthopaedics, where precision and personalized care are paramount, LLMs could play a transformative role [18].

For example, the global prevalence of osteoarthritis (OA) increased by 113.25 %, from 247.51 million cases in 1990 to 527.81 million in 2019 [19], with projections indicating further growth by 2030 [20]. An accessible and practical application of LLMs could involve preclinical patient selection for orthopaedic consultations. This includes identifying candidates for arthroplasty implantation, potentially conserving essential personnel and economic resources by streamlining straightforward OA diagnoses. Previous studies have already suggested ChatGPT's potential for intelligent question-answering in healthcare [21].

Building on the authors' expertise in hip and knee arthroplasty , as well as their previous research evaluating ChatGPT's diagnostic capabilities using physician-provided patient data [22], the present study aimed to further explore its diagnostic potential. The primary objective was to assess the sensitivity of ChatGPT in diagnosing OA of the hip and knee based solely on patient responses to a structured questionnaire, without medical interaction. Secondary objectives included comparing the diagnostic sensitivity of various ChatGPT models with other LLMs and identifying key factors contributing to accurate diagnoses across the models analysed.

## 2 Material and methods

### 2.1 Study design

A prospective observational clinical study was conducted at our orthopaedic clinic, focusing on patients diagnosed with hip or knee OA. To facilitate data collection, a pseudo-anonymized, paper-based questionnaire was specifically designed for this study and distributed to participants. This format was chosen to ensure accessibility for all participants, thereby minimizing potential barriers to engagement and promoting reliable data sampling.

The questionnaire comprised 18 questions across five categories: past medical history (5 questions), chief complaint and history of present illness (7 questions), family medical history (1 question), social history (3 questions), and additional information (2 questions).

The form was generated using GPT-4o based on a structured prompt tailored to meet the study’s objectives, ensuring the questionnaire adhered to a language and structure easily processed by LLMs. To validate its clinical relevance, a specialist in arthroplasty reviewed the survey, confirming its clarity and suitability for use in an orthopaedic setting.

In addition to the questionnaire, demographic data—age, weight, height, and sex—were recorded. A translated version of the original questionnaire is included as supplementary material, labelled "Questionnaire."

**Fig. 1:**
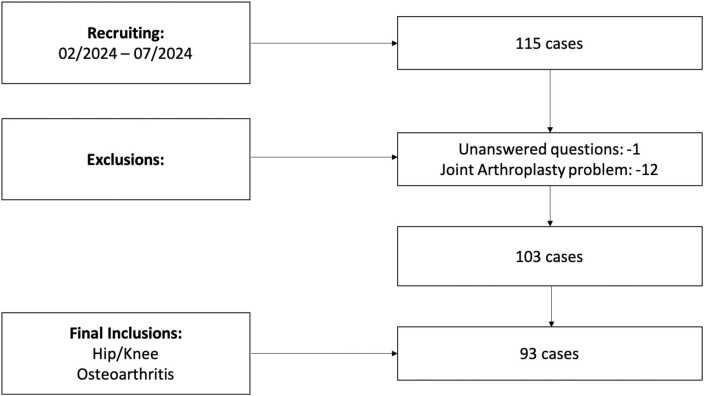
Flowchart of Patient Recruitment and Final Population Following Inclusion and Exclusion Criteria.

Eligible participants included individuals aged 18 years or older attending our specialized hip and knee OA clinic for the first time. Participants provided informed consent prior to inclusion. Recruitment occurred over seven clinic days from February to July 2024, yielding 115 completed questionnaires [Fig. 1].

The pseudo-anonymized questionnaires were collected prior to patient appointments. During the visits, experienced orthopaedic clinicians—blinded to the questionnaire content—performed clinical evaluations and provided diagnoses. Inclusion and exclusion criteria were applied based on the clinicians’ assessments.

Inclusion criteria comprised patients diagnosed with OA of the hip or knee, regardless of aetiology. Exclusion criteria included diagnoses unrelated to OA, consultations for non-OA-related issues (e.g., follow-up visits for previous surgeries), or incomplete responses to symptom-related questions concerning the chief complaint.

To maintain confidentiality, each participant was assigned a unique numeric code. This code was used solely to link questionnaire responses with corresponding medical records, enabling comparison between clinician and AI-generated diagnoses. Once the questionnaires were digitized, all identifiers, including numeric codes, were removed. De-identified data were then submitted to LLM models, which were prompted to generate diagnoses based exclusively on the questionnaire responses. The AI-generated diagnoses were subsequently compared with those made by the clinicians.

The clinical diagnosis served as the gold standard for evaluating the diagnostic performance of the models, given the straightforward nature of OA cases. A standardized approach was implemented to ensure consistency in the analysis. Each patient's responses, accompanied by a custom-designed prompt (details available in the supplementary material titled "Prompt"), were submitted to four ChatGPT models (GPT-4o, GPT-4 Turbo, GPT-4o Mini, GPT-3.5 Turbo), two Google Cloud LLMs (Gemini 1.5 Flash and Gemini 1.0 Pro), and three locally running LLMs (Llama-3.1, Gemma 2, and Mistral-Nemo). Further technical details regarding data processing are provided in the supplementary material titled "Data Processing Workflow."

### 2.2 Statistical analysis

For statistical analysis, key attributes of the questionnaire responses were evaluated, including total word count, word count specific to recent medical history questions (symptoms related to the chief complaint), the number of unanswered questions overall and within the recent medical history section, completeness (total responses provided out of the total number of questions), and detail (word count per response).

To assess and compare the diagnostic precision of the LLMs, sensitivity was calculated based on their ability to accurately identify OA diagnoses confirmed by clinicians, which served as the reference standard. Continuous variables are presented as means with standard deviations (SD), while categorical variables are reported as absolute numbers (n) and percentages (%). The Mann-Whitney *U* test was used to compare continuous variables between groups due to the non-normal distribution of the data.

Binary logistic regression analysis was employed to evaluate the relationship between response completeness/detail and the diagnostic accuracy of the models. Agreement between different LLMs was measured using Cohen’s Kappa coefficient [23].

Sample size was determined using power analysis for a single-test design for new diagnostic tools [24]. Assuming a type I error rate of 5 %, an expected diagnostic accuracy of at least 80 %, and an OA prevalence of at least 95 % in our preselected patient cohort, a minimum of 101 patients was required to achieve a maximum margin of error of 8 % with 95 % confidence [22], [25], [26], [27].

All statistical analyses were performed using IBM SPSS Statistics version 29.0, with statistical significance set at a two-sided p-value of < 0.05.

### 2.3 Ethical considerations

The study protocol was approved by the Ethics Committee of the University of Regensburg (Protocol Number 23–3590–101, January 2024). All participants provided informed consent, and the study was conducted in accordance with the Declaration of Helsinki.

## 3 Results

Of the 115 questionnaires initially collected, a total of 12 patients were excluded from the initial assessment. One patient was excluded for leaving all questions related to specific symptoms unanswered, while 11 patients were excluded as they sought treatment for issues related to joint prostheses.

Among the remaining 103 patients, 93 were diagnosed with OA of the hip or knee, while 10 were excluded due to conditions unrelated to the study's primary focus. For detailed demographic characteristics and diagnoses, please refer to Table 1.

**Table 1:** Demographic Characteristics of the Study Population. The table includes a quantitative and qualitative analysis of patient responses, examining the number of words per response and the frequency of non-responses (NA). The term “completeness” refers to the proportion of questions answered, while “detail” is defined as the average word count per response.

	**Total (n = 93)**
**Age (y)**	
Range	38–85
Mean ( ± SD)	65.1 ( ± 9.7)
**Sex**	
Female	51 (54.8 %)
Male	42 (45.2 %)
**BMI**	
Range	17.8–46.0
Mean ( ± SD)	29.3 ( ± 5.3)
**Affected Joint**	
Knee	42 (45.2 %)
Hip	51 (54.8 %)
**Words (n)**	
Range	20 – 103
Mean ( ± SD)	55.7 ( ± 19.1)
**NA (n)**	
Range	0 – 12
Mean ( ± SD)	3.4 ( ± 3.5)
**Completeness (%)**	
Range	33.3 – 100 %
Mean ( ± SD)	81.3 % ( ± 19.3)
**Detail (n)**	
Range	1.3 – 11.4
Mean ( ± SD)	4.5 ( ± 2.0)

Y: years, SD: Standard Deviation, NA: not answered questions

### 3.1 Diagnostic sensitivity of LLMs

**Fig. 2:**
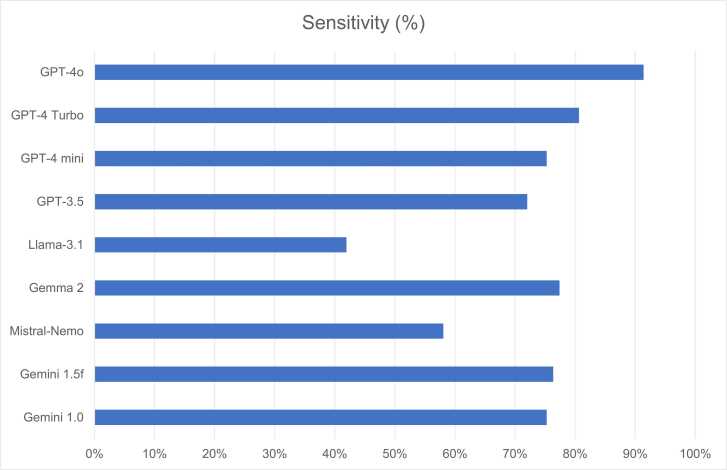
Sensitivity of LLMs in Diagnosing OA. This graphic provides a comparative analysis of diagnostic sensitivity across the LLMs. GPT-4o and GPT-4 Turbo demonstrated the highest accuracy, while Llama-3.1 and Mistral-Nemo showed comparatively lower performance.

In terms of model sensitivity, GPT-4o demonstrated the highest performance, achieving a diagnostic sensitivity of 92.3 %. By contrast, Llama-3.1 recorded the lowest sensitivity, at 42.9 % (Fig. 2). When analysing hip and knee OA separately, GPT-4o again outperformed other models, with a sensitivity of 86.3 % for hip OA (51 cases) and 97.6 % for knee OA (42 cases).

### 3.2 Impact of response completeness on diagnostic accuracy

A univariate analysis was conducted to evaluate the influence of response completeness on model performance. For GPT-4o, the completeness of responses was a significant predictor of diagnostic accuracy, both for overall responses (p = 0.014) and symptom-specific responses (p < 0.001). Significant associations were also observed for Gemini 1.5 (p = 0.017) and Gemini 1.0 (p = 0.012), as detailed in Table 2.

**Table 2:** Comparison Between Response Completeness, Detail, and Diagnostic Accuracy of LLMs. Highlights the relationship between response completeness, detail, and diagnostic accuracy for each model, considering both the entire questionnaire and symptom-specific questions.

		**Total questions (n = 18)**				**Symptom related questions (n = 7)**			
	**Diagnosis (n,a)**	**Words (m,SD)**	**NA (m,SD)**	**Completeness (m,SD)**	**Detail (m,SD)**	**Words (m,SD)**	**NA (m,SD)**	**Completeness (m,SD)**	**Detail (m,SD)**
GPT−4o									
Correct	85 (91.4 %)	55.5 ± 19.2	3.5 ± 3.4	80.5 ± 19.0 %	3.1 ± 1.1	31.1 ± 14.3	0.5 ± 0.9	92.6 ± 13.2 %	4.4 ± 2.0
Wrong	8 (8.6 %)	58.3 ± 18.0	1.9 ± 3.9	89.6 ± 21.9 %	3.2 ± 1.0	32.6 ± 10.5	1.9 ± 1.5	73.2 ± 20.8 %	4.7 ± 1.5
p	-	0.429	**0.014**	**0.014**	0.429	0.375	**< 0.001**	**< 0.001**	0.375
GPT−4 Turbo									
Correct	75 (80.6 %)	55.4 ± 18.9	3.4 ± 3.5	81.1 ± 19.2 %	3.1 ± 1.1	30.6 ± 13.2	0.5 ± 0.9	92.6 ± 13.4 %	4.4 ± 1.9
Wrong	18 (19.4 %)	57.1 ± 20.4	3.2 ± 3.7	82.1 ± 20.4 %	3.2 ± 1.1	33.7 ± 16.9	1.1 ± 1.3	84.1 ± 18.9 %	4.8 ± 2.4
p	-	0.931	0.763	0.763	0.931	0.641	0.063	0.063	0.641
GPT−4 mini									
Correct	70 (75.3 %)	56.0 ± 19.3	3.2 ± 3.2	82.3 ± 17.8 %	3.1 ± 1.1	31.3 ± 14.6	0.5 ± 0.9	92.3 ± 13.5 %	4.5 ± 2.1
Wrong	23 (24.7 %)	54.8 ± 18.6	3.9 ± 4.2	78.3 ± 23.5 %	3.0 ± 1.0	30.9 ± 11.9	0.9 ± 1.3	87.0 ± 18.2 %	4.4 ± 1.7
p	-	0.910	0.826	0.826	0.910	0.732	0.150	0.150	.732
GPT−3.5									
Correct	67 (72.0 %)	54.3 ± 17.3	3.0 ± 3.2	83.4 ± 18.0 %	3.0 ± 1.0	30.3 ± 13.0	0.6 ± 1.0	90.8 ± 14.7 %	4.3 ± 1.9
Wrong	26 (28.0 %)	59.3 ± 22.9	4.3 ± 3.9	75.9 ± 21.9 %	3.3 ± 1.3	33.7 ± 16.1	0.6 ± 1.1	91.2 ± 15.7 %	4.8 ± 2.3
p	-	0.358	0.239	0.239	0.358	0.294	0.960	0.960	0.294
Llama−3.1									
Correct	39 (41.9 %)	55.3 ± 17.5	3.5 ± 3.4	80.6 ± 19.0 %	3.1 ± 1.0	29.1 ± 12.8	0.3 ± 0.6	96.0 ± 8.6 %	4.2 ± 1.8
Wrong	54 (58.1 %)	56.1 ± 20.3	3.3 ± 3.5	81.8 ± 19.7 %	3.1 ± 1.1	32.8 ± 14.7	0.9 ± 1.2	87.3 ± 17.3 %	4.7 ± 2.1
p	-	0.976	0.524	0.524	0.976	0.239	**0.007**	**0.007**	0.239
Gemma 2									
Correct	72 (77.4 %)	55.9 ± 18.9	3.3 ± 3.4	81.7 ± 18.8 %	3.1 ± 1.1	31.4 ± 14.5	0.6 ± 1.0	91.9 ± 14.4 %	4.5 ± 2.1
Wrong	21 (22.6 %)	55.0 ± 20.0	3.6 ± 3.9	79.9 ± 21.6 %	3.1 ± 1.1	30.8 ± 12.1	0.9 ± 1.1	87.8 ± 16.5 %	4.4 ± 1.7
p	-	0.904	0.793	0.793	0.904	0.813	0.095	0.095	0.813
Mistral-Nemo									
Correct	54 (58.1 %)	55.1 ± 17.6	3.5 ± 3.5	80.6 ± 19.5 %	3.1 ± 1.0	30.1 ± 13.4	0.5 ± 1.0	92.9 ± 13.8 %	4.3 ± 1.9
Wrong	39 (41.9 %)	56.5 ± 21.1	3.2 ± 3.5	82.3 ± 19.3 %	3.1 ± 1.2	32.8 ± 14.8	0.8 ± 1.1	88.3 ± 16.0 %	4.7 ± 2.1
p	-	0.924	0.543	0.543	0.924	0.355	0.085	0.085	0.355
Gemini 1.5									
Correct	71 (76.3 %)	56.1 ± 19.5	3.4 ± 3.5	81.1 ± 19.5 %	3.1 ± 1.1	31.4 ± 14.7	0.5 ± 0.9	92.8 ± 13.4 %	4.5 ± 2.1
Wrong	22 (23.7 %)	54.6 ± 18.1	3.2 ± 3.5	82.1 ± 19.2 %	3.0 ± 1.0	30.6 ± 11.5	1.1 ± 1.3	85.1 ± 17.9 %	4.4 ± 1.6
p	-	0.916	0.705	0.705	0.916	0.829	**0.017**	**0.017**	0.829
Gemini 1.0									
Correct	70 (75.3 %)	57.0 ± 19.1	3.3 ± 3.5	81.6 ± 19.3 %	3.2 ± 1.1	32.1 ± 14.7	0.5 ± 0.9	93.1 ± 13.5 %	4.6 ± 2.1
Wrong	23 (24.7 %)	51.9 ± 18.9	3.5 ± 3.6	80.4 ± 19.9 %	2.9 ± 1.1	28.5 ± 11.4	1.1 ± 1.2	84.5 ± 17.2 %	4.1 ± 1.6
p	-	0.190	0.752	0.752	0.190	0.349	**0.012**	**0.012**	0.349

a: accuracy, m: mean, SD: standard deviation, NA: not answered. Bold means significant two-sided p (p < 0.05)

In the multivariate analysis, the importance of response completeness remained significant. For GPT-4o, symptom response completeness remained a significant factor (p = 0.002, 95 % CI Exp(B) = 1.072–1.119). Similarly, significant associations were observed for Gemini 1.5 (p = 0.021, 95 % CI Exp(B) = 1.005–1.067) and Gemini 1.0 (p = 0.034, 95 % CI Exp(B) = 1.002–1.063). Conversely, Llama-3.1, which had the lowest diagnostic accuracy, was more strongly affected by the total number of unanswered questions (p = 0.006, 95 % CI Exp(B) = 0.271–0.808).

### 3.3 Inter-model agreement

Inter-model agreement was assessed using Cohen’s Kappa. GPT-4o, GPT-4 Turbo, and GPT-4 mini exhibited moderate concordance, with Kappa values exceeding 0.40. In contrast, Llama-3.1 and GPT-3.5 demonstrated weaker agreement with other models, with Kappa values ranging between 0.13 and 0.22. The Gemini models (1.5 and 1.0) displayed particularly strong concordance with each other, achieving a Kappa value of 0.597 [28] (Table 3).

**Table 3:** Inter-Rater Reliability Comparison Expressed Through Cohen’s Kappa. This table outlines inter-rater reliability among the LLMs. Values below 0.2 indicate minimal agreement, 0.21–0.39 suggest weak agreement, and 0.40–0.59 indicate moderate agreement [23], [28]. No model achieved a Kappa value ≥ 0.6, which signifies strong agreement. The p-values associated with each comparison are presented in parentheses.

**LLM**	**GPT-4o**	**GPT-4Turbo**	**GPT-4mini**	**GPT-3.5**	**Llama-3.1**	**Gemma 2**	**Mistral-Nemo**	**Gemini 1.5**	**Gemini 1.0**
GPT−4o	1	0.448 (<0.001)	0.401 (<0.001)	0.222 (0.008)	0.140 (0.008)	0.365 (<0.001)	0.250 (<0.001)	0.348 (<0.001)	0.275 (0.002)
GPT−4Turbo	0.448 (<0.001)	1	0.512 (<0.001)	0.336 (<0.001)	0.134 (0.065)	0.430 (<0.001)	0.297 (<0.001)	0.534 (<0.001)	0.410 (<0.001)
GPT−4mini	0.401 (<0.001)	0.512 (<0.001)	1	0.382 (<0.001)	0.234 (0.004)	0.425 (<0.001)	0.403 (<0.001)	0.404 (<0.001)	0.348 (<0.001)
GPT−3.5	0.222 (0.008)	0.336 (<0.001)	0.382 (<0.001)	1	0.167 (0.052)	0.422 (<0.001)	0.341 (<0.001)	0.184 (0.072)	0.130 (0.204)
Llama−3.1	0.140 (0.008)	0.134 (0.065)	0.234 (0.004)	0.167 (0.052)	1	0.122 (0.122)	0.564 (<0.001)	0.256 (0.001)	0.295 (<0.001)
Gemma 2	0.365 (<0.001)	0.430 (<0.001)	0.425 (<0.001)	0.422 (<0.001)	0.122 (0.122)	1	0.353 (<0.001)	0.387 (<0.001)	0.328 (0.001)
Mistral-Nemo	0.250 (<0.001)	0.297 (<0.001)	0.403 (<0.001)	0.341 (<0.001)	0.564 (<0.001)	0.353 (<0.001)	1	0.470 (<0.001)	0.470 (<0.001)
Gemini 1.5	0.348 (<0.001)	0.534 (<0.001)	0.404 (<0.001)	0.184 (0.072)	0.256 (0.001)	0.387 (<0.001)	0.470 (<0.001)	1	0.597 (<0.001)
Gemini 1.0	0.275 (0.002)	0.410 (<0.001)	0.348 (<0.001)	0.130 (0.204)	0.295 (<0.001)	0.328 (0.001)	0.470 (<0.001)	0.597 (<0.001)	1

## 4 Discussion

In this study, GPT-4o demonstrated high diagnostic sensitivity, achieving an impressive rate of 92.3 %, which is comparable to prior research involving ChatGPT-4 that reported a 100 % correct diagnostic rate in OA cases [22]. However, key methodological differences between the two studies should be noted. The earlier study supplied the language model with comprehensive clinical data, including physician notes, physical examination findings, and radiographs, whereas this study relied solely on patient-completed questionnaires without input from healthcare professionals. Despite this limitation, GPT-4o’s performance underscores its diagnostic potential.

The primary objective of this study was to evaluate GPT-4o's diagnostic capabilities in the absence of direct patient interaction or medical oversight. Unlike in-person assessments where physicians can address ambiguities or incomplete information, GPT-4o operated without real-time feedback. While the study does not suggest that the model can replace physicians, it highlights GPT-4o’s promise as an adjunct tool to support clinical workflows and enhance diagnostic processes.

GPT-4o’s high sensitivity suggests its potential utility in preliminary triage, particularly in busy clinical environments where rapid assessment could expedite specialist consultations. For example, GPT-4o could guide patients toward appropriate care pathways, especially when surgical interventions like joint replacement are under consideration. This aligns with prior studies highlighting the utility of LLMs in clinical decision-making [15], [29].

Kunze et al. demonstrated that ChatGPT-4 could effectively triage common knee pain complaints by generating concise differential diagnoses within appropriate clinical contexts. When additional patient details, such as age and medical history, were included, ChatGPT-4 achieved a diagnostic accuracy of 100 %, aligning closely with the findings of this study [26]. Expanding the application of ChatGPT in triage, Kaboudi et al. conducted a meta-analysis evaluating 14 studies with a total of 1412 patients or scenarios. They found that ChatGPT-4, with a pooled accuracy of 0.86 (95 % CI: 0.64–0.98), outperformed ChatGPT-3.5, which had an accuracy of 0.63 (95 % CI: 0.43–0.81) [27].

This study’s findings align with prior research regarding the relative performance of different language models. For instance, GPT-4o consistently outperformed Llama-3.1 in diagnostic sensitivity, likely due to its advanced architecture and computational capacity. Sandmann et al. demonstrated a similar trend, observing declining accuracy across GPT-4, GPT-3.5, and Llama-3.1 in analysing 110 clinical cases [30]. GPT-4o’s enterprise-grade infrastructure enables it to handle larger datasets and implement proprietary algorithms that enhance diagnostic precision. In contrast, Llama-3.1’s general-purpose design and limited computational resources hinder its performance in specialized medical tasks [7], [30].

Moreover, GPT-4o demonstrated superior performance compared to Gemini Advanced and GPT-4 in interpreting ECGs, showing moderate agreement with GPT-4 and weaker agreement between GPT-4 and Gemini—a trend consistent with this study’s results [31]. GPT-4o also surpassed Gemini 1.5 in diagnostic accuracy in clinical radiology case quizzes, underscoring its versatility across diagnostic domains

[32].

The findings also align with observations from other medical specialties and more complex clinical cases and diagnoses. For example, Gemini Advanced achieved 81.87 % accuracy in gynaecologic oncology decision-making tasks [33], while a study in oral surgery reported a 71.7 % accuracy rate for ChatGPT-4. These results suggest that LLMs can function as intelligent virtual assistants, complementing rather than replacing clinical expertise [34].

A critical insight from this study is the importance of response completeness, particularly for symptom-specific queries, in influencing GPT-4o’s diagnostic accuracy. This highlights the value of detailed patient interactions and suggests that optimizing data collection processes could further enhance the diagnostic potential of LLMs in healthcare.

However, the integration of AI-driven models into clinical practice raises complex ethical considerations. Issues surrounding patient autonomy, confidentiality, and the potential for misdiagnosis demand careful oversight. Future implementation strategies should emphasize expert validation of AI outputs, robust data protection measures (e.g., anonymization and encryption), and a collaborative “co-pilot” approach that maintains clinicians’ decision-making authority. Regulatory guidelines, transparency in AI usage, and iterative refinements informed by real-world validation studies will be crucial to uphold ethical standards [35], [36].

LLMs, including GPT-4o, are best positioned as support tools that streamline workflows, such as triage or preliminary assessments, while preserving the irreplaceable expertise and empathy of healthcare professionals [37], [38], [39].

This study’s novel design is among the first prospective, real-world investigations of diagnostic sensitivity in both commercial and non-commercial LLMs within medical and orthopaedic fields. Nonetheless, several limitations should be acknowledged. The reliance on patient-provided questionnaires without direct medical input limited the depth and accuracy of the information, potentially affecting the models’ diagnostic performance. Additionally, the use of a paper-based questionnaire restricted patient interaction with the model, which could have been enhanced by a digital chatbot allowing for more detailed responses or clarifications. The relatively small sample size of 93 patients may have reduced the statistical power and limited the generalizability of the findings. The exclusion of patients with complex diagnoses or joint prostheses further narrowed the study’s scope. Furthermore, the diagnostic performance of language models is heavily influenced by prompt quality, suggesting that future research should prioritize refining prompt engineering [40].

Technical challenges also precluded the inclusion of Microsoft Copilot (Microsoft Corporation, Redmond, WA, USA), a widely used LLM. Factors such as limited API compatibility and high subscription costs associated with Microsoft Azure infrastructure rendered its evaluation impractical within this study’s resource constraints [41]. Future studies should consider incorporating a broader range of models, including Microsoft Copilot, to enhance comprehensiveness.

Finally, while GPT-4o has demonstrated advancements in handling non-English languages, as noted in recent updates from OpenAI [3], subtle linguistic or cultural nuances in patient responses could still affect its diagnostic accuracy in non-English contexts. Furthermore, the study did not account for emotional, psychological, or cognitive factors that may influence the quality of patient responses, further limiting the external validity of its findings.

## 5 Conclusion

This study demonstrates that GPT-4o can achieve a high diagnostic sensitivity in hip and knee OA using solely patient-reported questionnaires. While GPT-4o outperformed other LLMs, response completeness was a critical factor in its performance. Despite its effectiveness, the lack of direct patient interaction underscores the necessity of physician oversight, particularly in ensuring the safe and reliable integration of GPT-4o as a supplementary tool in preclinical settings.

Future research should explore its applicability to more complex orthopaedic cases, such as revision arthroplasty, and evaluate its integration into specialized clinical workflows. Refining data collection processes, optimizing prompt engineering, and incorporating real-time patient feedback will be key to expanding the model’s diagnostic utility while addressing its current limitations

## Originality and Ethical Compliance

o The manuscript represents original work and has not been published elsewhere, nor is it under consideration for publication elsewhere.
o Any previously published data, text, or figures have been appropriately cited and referenced.
o All ethical considerations, including those involving human or animal subjects, have been adhered to in accordance with institutional and international guidelines. Ethical approval and patient consent (where applicable) have been obtained and are explicitly stated in the manuscript.

## Declaration of Generative AI and AI-assisted technologies in the writing process

During the preparation of this manuscript, the authors utilized ChatGPT Version 4o by OpenAI exclusively for grammar correction and refinement of the text style in the original draft. The authors thoroughly reviewed and edited the content following its use and take full responsibility for the final version of the publication.

## Conflict of Interest

All authors declare no financial or non-financial competing interests.
