# Supplementary material for: Evaluating ChatGPT, Gemini and other Large Language Models (LLMs) in orthopaedic diagnostics: A prospective clinical study
Source: Comput Struct Biotechnol J. 2024 Dec 26;28:9–15. doi: 10.1016/j.csbj.2024.12.013 (PMC11754967; doi:10.1016/j.csbj.2024.12.013)
Supplement: Supplementary file 1 — Supplementary material [file mmc1.zip › Supplemental Material/Data Processing Workflow.docx]

## Data Processing Workflow and Performance Evaluation of Large Language Models (LLMs) in Diagnostic Queries

Structured Query Language (SQL) was employed to manage data, with SQLite chosen as the primary database management system due to its simplicity, compatibility with Google Drive, and efficient query execution.

The initial dataset, derived from patient questionnaires, was compiled into an Excel file for organization and easy access.

Data was transformed and transferred into the database using Extract, Transform, Load (ETL) tools, specifically Pentaho Data Integration (PDI). All programming tasks were performed in Python, with Google Colab used as the primary coding environment and Jupyter Notebook as a local alternative.

An Application Programming Interface (API) played a key role in this workflow, enabling efficient querying across different services while ensuring data consistency.

**Prompt Configuration**

The diagnostic prompt was crafted to elicit relevant information without extraneous details. It contained 2,918 characters, using 674 tokens per query. A temperature setting of 0.4 was applied uniformly to maintain consistent responses.

**Data Processing Workflow**

The data processing workflow followed these steps:

1. Sending a system prompt containing individual patient data to the model.
2. Storing the model's output in the designated database column.
3. Resetting variables and repeating the process for subsequent entries.

**Models Utilized**

Nine different large language models (LLMs) were employed in this study:

- GPT-4o (OpenAI, San Francisco , California, US)
- GPT-4 Turbo (OpenAI, San Francisco , California, US)
- GPT-4o Mini (OpenAI, San Francisco , California, US)
- GPT-3.5 Turbo (OpenAI, San Francisco , California, US)
- Gemini-1.5 Flash (Google, Alphabet, Mountain View, California, US)
- Gemini-1.0 Pro (Google, Alphabet, Mountain View, California, US)
- Llama 3.1 (Meta, Menlo Park, California, US)
- Gemma 2 (Google, Alphabet, Mountain View, California, US)
- Mistral-Nemo (Mistral AI, Paris, France)

Please refer to Table 1 for detailed technical specifications.

Table 1 – Technical Specifications of the Chatbots used in the Study. The table highlights key details such as model size, which directly correlates with the model's data capacity and its ability to generate complex and accurate responses. For models operating on local hardware, the specifications of the computer used during testing are listed. The token limit indicates the approximately amount of information the chatbot can process and retain from the input prompt per query. Detailed references for each model are included to provide further context.

| **Model Name** | **Ownership** | **Model Size** | **Hardware** | **Memory** | **Token Limit** | **Reference** |
| --- | --- | --- | --- | --- | --- | --- |
| GPT-4 Turbo | OpenAI | ~100T | Cloud-based | - | 32,768 | [OpenAI Release Notes](https://help.openai.com/) |
| GPT-4o | OpenAI | ~1.7T | Cloud-based | - | 16,384 | [OpenAI Release Notes](https://cdn.openai.com/gpt-4o-system-card.pdf) |
| GPT-4o Mini | OpenAI | ~15B | Cloud-based | - | 16,384 | [OpenAI Documentation](https://openai.com/index/gpt-4o-mini-advancing-cost-efficient-intelligence/) |
| GPT-3.5 Turbo | OpenAI | ~175B | Cloud-based | - | 4,096 | [OpenAI Documentation](https://openai.com/index/gpt-3-5-turbo-fine-tuning-and-api-updates/) |
| Gemini 1.5 Flash | Google | ~32B | Cloud-based | - | 8,192 | [Google AI Documentation](https://deepmind.google/technologies/gemini/) |
| Gemini 1.0 Pro | Google | ~8B | Cloud-based | - | 8,192 | [Google AI Documentation](https://deepmind.google/technologies/gemini/) |
| Llama 3.1 | Meta | ~8B | Local - NVIDIA RTX 2060 GPU | 16 GB RAM, 6 GB VRAM | 4,096 | [Meta Llama Release](https://ai.meta.com/static-resource/responsible-use-guide/) |
| Gemma 2 | Independent | ~9B | Local - NVIDIA RTX 2060 GPU | 16 GB RAM, 6 GB VRAM | 8,192 | [Gemma models overview](https://ai.google.dev/gemma/docs) |
| Mistral-Nemo | Independent | ~12B | Local - NVIDIA RTX 2060 GPU | 16 GB RAM, 6 GB VRAM | 4,096 | [Mistral documentation](https://docs.mistral.ai/) |
| T: Trillion parameters, B: Billion parameters, RAM: Random Access Memory, VRAM: Video Random Access Memory, GPU: Graphics Processing Unit | | | | | | |

**Cloud-Based LLMs**

The analysis focused on two primary platforms: OpenAI’s ChatGPT and Google’s Gemini. Both platforms demonstrated low response times, typically under one second, with rare instances exceeding three seconds, largely influenced by connection speed. OpenAI’s GPT-3.5 Turbo occasionally produced verbose, off-topic responses. In contrast, GPT-4o proved to be more token-efficient. Google's Gemini-1.0 Pro model did not support the "system_instruction" parameter, so a single request prompt was used for consistency with the Gemini-1.5 Flash model. A key advantage of the Gemini models is their no-cost plan, provided usage limits are not exceeded.

**Local LLMs**

Local models presented the advantage of no usage restrictions, though response times were dependent on hardware. The setup included an NVIDIA GeForce RTX 2060 mobile GPU with CUDA drivers, which handled the processing without overloading, averaging around 35% usage.

Average response times for the local models were as follows:

- Llama 3.1: approximately 13 seconds
- Gemma 2: approximately 18 seconds
- Mistral-Nemo: approximately 22 seconds

Disk space usage for each model was:

- Llama 3.1: approximately 4.7 GB
- Gemma 2: approximately 5.4 GB
- Mistral-Nemo: approximately 7.1 GB

The local models were accessed through the Ollama API, which facilitated model retrieval and execution. Methodologies and insights on class and function development were guided by the book "Hands-On Large Language Models" by O'Reilly [1].

**References**

1. Alammar, J. (2024). *Hands-on large language models: Language understanding and generation.* O'Reilly Media.
