# Supplementary material for: Evaluating ChatGPT, Gemini and other Large Language Models (LLMs) in orthopaedic diagnostics: A prospective clinical study
Source: Comput Struct Biotechnol J. 2024 Dec 26;28:9–15. doi: 10.1016/j.csbj.2024.12.013 (PMC11754967; doi:10.1016/j.csbj.2024.12.013)
Supplement: Supplementary file 1 — Supplementary material [file mmc1.zip › Supplemental Material/Prompt.docx]

I am working on a scientific research project in the field of orthopaedics and would like to optimally utilize the functions of ChatGPT-4. The goal is to evaluate the capability of ChatGPT-4 to provide a diagnosis in the orthopaedic field based on demographic data and a patient's responses to a questionnaire.

Please analyse the information I provide and give the most likely and accurate diagnosis:

**Patient's Demographic Data:**

- Height, Weight, Age, Gender (M:0, F:1)

**Questionnaire:**

**Medical History**

1. Relevant pre-existing conditions (e.g., hypertension, rheumatism, diabetes, heart attack, stroke):
2. Previous orthopaedic diseases or injuries:
3. Current medications (please specify active ingredients only):
4. Known allergies:
5. Previous orthopaedic surgeries:

**Specific Symptoms** 6. Description of pain (type, intensity, duration, variability): 7. Location of pain or discomfort (exact spot, radiation): 8. Onset of symptoms and their development (e.g., after an accident): 9. Activities or movements that aggravate or relieve the pain: 10. Presence of swelling, redness, or warmth: 11. Limitations in mobility or daily activities: 12. Presence of numbness, tingling, or other sensory abnormalities:

**Lifestyle and Habits** 13. Occupational activities (type and degree of physical strain): 14. Sporting activities (type, frequency, intensity): 15. Smoking and alcohol consumption (quantity and frequency):

**Family History** 16. Presence of orthopaedic diseases in the family:

**Additional Notes** 17. Special life circumstances or stress factors that could influence the symptoms: 18. Other relevant information or symptoms not queried:

**Goal:** Based on the following patient responses from an orthopaedic questionnaire, please provide the most likely preliminary diagnosis for research purposes. Analyze these responses and provide the most likely orthopedic preliminary diagnosis based on the symptomatology and patient's history.
