# Supplementary material for: Evaluating ChatGPT, Gemini and other Large Language Models (LLMs) in orthopaedic diagnostics: A prospective clinical study
Source: Comput Struct Biotechnol J. 2024 Dec 26;28:9–15. doi: 10.1016/j.csbj.2024.12.013 (PMC11754967; doi:10.1016/j.csbj.2024.12.013)
Supplement: Supplementary file 1 — Supplementary material [file mmc1.zip › Supplemental Material/Questionnaire.docx]

**Orthopaedic Patient Questionnaire (Pseudonymous)**

**General Information**

Case Number: _______________     Date: ___________________

Height: ____________________      Weight: ___________________

Age: _____________________       Gender: ________________

**Medical History**

1. **Relevant pre-existing conditions** (e.g., high blood pressure, rheumatism, diabetes, heart attack, stroke):
2. **Previous orthopaedic diseases or injuries**:
3. **Current medications** (please list active ingredients only):
4. **Known allergies**:
5. **Previous orthopaedic surgeries**:

**Specific Symptoms**

1. **Description of the pain** (type, intensity, duration, variability):
2. **Location of the pain or discomfort** (exact spot, radiation):
3. **Time of first onset of symptoms and their development** (e.g., after an accident):
4. **Activities or movements that aggravate or alleviate the pain**:
5. **Presence of swelling, redness, or warmth**:
6. **Limitations in mobility or daily activities**:
7. **Presence of numbness, tingling, or other sensory abnormalities**:

**Lifestyle and Habits**

1. **Occupational activities** (type and degree of physical strain):
2. **Sporting activities** (type, frequency, intensity):
3. **Smoking and alcohol consumption** (amount and frequency):

**Family Medical History**

1. **Presence of orthopaedic diseases in the family**:

**Additional Notes**

1. **Special life circumstances or stress factors that could influence the symptoms**:
2. **Further relevant information or symptoms that were not inquired about**:
